# Transcriptional Regulation of Programmed Hypertension by Melatonin: An Epigenetic Perspective

**DOI:** 10.3390/ijms151018484

**Published:** 2014-10-14

**Authors:** You-Lin Tain, Li-Tung Huang, Julie Y. H. Chan

**Affiliations:** 1 Departments of Pediatrics, Kaohsiung Chang Gung Memorial Hospital and Chang Gung University College of Medicine, Kaohsiung 833, Taiwan; E-Mail: litung.huang@gmail.com; 2 Center for Translational Research in Biomedical Sciences, Kaohsiung Chang Gung Memorial Hospital and Chang Gung University College of Medicine, Kaohsiung 833, Taiwan; E-Mail: jchan@adm.cgmh.org.tw; 3 Department of Traditional Chinese Medicine, Chang Gung University, Linkow 244, Taiwan

**Keywords:** developmental programming, epigenetic regulation, hypertension, melatonin, next generation sequencing, oxidative stress, renin-angiotensin system

## Abstract

Melatonin is an endogenously produced indoleamine and secreted by the pineal gland. Melatonin has pleiotropic bioactivities and is involved in epigenetic regulation. Suboptimal conditions during maternal and perinatal phases can elicit epigenetic regulation of genes for nephrogenesis and reset physiological responses to develop programmed hypertension. This review discusses the early utility of melatonin to prevent programmed hypertension in later life by epigenetic regulation in the kidney, with an emphasis on: (1) the role of melatonin in epigenetic regulation; (2) the beneficial effects of melatonin on programmed hypertension; (3) epigenetic regulation of maternal melatonin therapy in different developmental windows of offspring kidneys analyzed by whole-genome RNA next-generation sequencing; and (4) current blocks in the application of melatonin in preventing programmed hypertension.

## 1. Introduction

Hypertension may originate during early life. Suboptimal conditions during maternal and perinatal phases can elicit epigenetic alterations in genes involved in organogenesis, morphological changes and adaptive physiological responses, namely developmental programming [1,2]. The most common outcome is programmed hypertension [1,2]. We recently observed that programmed hypertension developed in the male offspring of rats exposed to a variety of insults, including maternal caloric restriction [3,4], diabetes [5], high fructose (HF) diet [6] and dexamethasone (DEX) treatment [7,8]. Many mechanisms, including glucocorticoid effects, oxidative stress, epigenetic regulation, alterations of the renin-angiotensin system (RAS) and reduction in nephron numbers, have been proposed to interpret the programming of hypertension, but none have received recognition [1,2]. The kidney is an important organ for long-term blood pressure (BP) regulation, is particularly susceptible to the insults of programming during nephrogenesis and has been identified as a key player in programmed hypertension [9]. In both human and experimental hypertension, we and others have demonstrated some particular candidate genes and pathways in the kidney related to programmed hypertension, including nephrogenesis, oxidative stress, epigenetic regulation, RAS and sodium transporters [1,2,3,4,5,6,7,8,9].

Melatonin (*N*-acetyl-5-methoxytryptamine) is an endogenously produced indoleamine of the pineal gland. It has been shown to have antioxidant and anti-inflammatory properties, and it can scavenge free radicals, as well as control the circadian rhythm [10,11]. We previously reported that melatonin confers protection against oxidative stress and hypertension in young spontaneously hypertensive rats and in some models of programmed hypertension [4,6,12]. In addition to its antioxidant properties, emerging evidence indicates the importance of melatonin in epigenetic modulation [13]. In our subsequent studies, we demonstrated that epigenetic regulation by melatonin is related to the prevention of programmed hypertension [4,7].

Although melatonin has been shown to restore the redox status to treat adverse programming effects associated with compromised pregnancies [14], the potential role of melatonin in epigenetic modifications remains unclear. Epigenetic mechanisms play a critical role during placental maturation, organogenesis and development [15]. However, organs do not react in the same manner to developmental programming, leading to organ-specific epigenetic modifications of gene cascades. This review is a modest attempt to summarize and discuss the current state of research on the epigenetic regulation of melatonin in programmed hypertension, with special emphasis on the kidney.

## 2. Role of Melatonin in Epigenetic Regulation

Epigenetics refers to alterations in gene expression that are not explained by changes in DNA sequence. DNA methylation, histone modification and RNA interference play central roles in epigenetic regulation [16]. DNA methyltransferases (DNMTs) are a family of enzymes that methylate DNA, and they play a crucial role in epigenetic regulation. Interestingly, melatonin has a similar structure, and it can hypothetically inhibit DNMT by either masking target sequences or blocking the active site of the enzyme [17]. While DNA methylation is relatively permanent and results in the silencing of genes, the modification of histone tails has been considered to be more responsive to the nutritional and environmental insults occurring during the programming process [18]. Histone acetylation is one of the most frequent epigenetic modifications. Histone acetyltransferases (HATs) and histone deacetylases (HDACs) determine histone acetylation and deacetylation, respectively. Our recent work suggests that early melatonin therapy administered to the mother rat may elicit epigenetic changes in the kidney of the offspring, leading to long-term amelioration of hypertension [4]. We found that melatonin up-regulated the expression of HDAC-2, HDAC-3 and HDAC-8 in the kidneys of calorie-restricted (CR) offspring treated with melatonin. This finding is consistent with a previous finding that melatonin increased the expression of both class I and class II HDACs *in vitro* [19]. Conversely, melatonin is known to be a class III HDAC inhibitor [20]. This is consistent with our recent finding that melatonin-prevented neonatal DEX exposure induced the increases of HDAC 1–3 proteins and programmed hypertension [21]. Furthermore, these changes were similar in response to melatonin therapy, as well as in response to trichostatin A (TSA, HDAC inhibitor) treatment. These findings support the possibility that melatonin may act as an HDAC inhibitor to protect against the development of hypertension in neonatal DEX-exposed rats. Given that HDACs are thought to repress gene, melatonin is liable to induce gene expression. This is supported by our recent findings that maternal melatonin therapy increases the expression of >400 genes in the developing kidney in a CR-induced programmed hypertension model [4]. Thus, melatonin might have dual effects on HDACs to regulate gene expression differentially.

## 3. Beneficial Effects of Melatonin on Programmed Hypertension

Melatonin has pleiotropic bioactivities and is involved in the regulation of the circadian rhythm, reproductive physiology, antioxidant and anti-inflammatory responses, mitochondrial biogenesis and prevention of tumor progression [10,11]. Emerging evidence indicates that melatonin is beneficial to reverse the adverse programming effects associated with compromised pregnancies, including diabetes, metabolic syndrome, maternal malnutrition, preeclampsia and the effects of dexamethasone exposure [14,22,23,24]. Despite evidence from human and experimental studies showing antihypertensive effects of melatonin on established hypertension [25,26,27], so far, few data are available regarding the protective effects of melatonin on programmed hypertension.

Our recent work demonstrated that the protective effects of melatonin may not be identical in different models of programmed hypertension. In a 50% CR model, maternal melatonin therapy prevented CR-induced programmed hypertension related to the restoration of nitric oxide (NO), alteration of RAS and epigenetic changes in numerous genes [4]. Additionally, we found melatonin attenuated programmed hypertension in a prenatal DEX-exposure model by restoration of nephron numbers, alteration of RAS components and modulation of HDACs [7]. In a maternal high-fructose (HF) intake model, the beneficial effects of melatonin are due to its ability to increase NO level, epigenetic regulation of genes related to BP control and inhibition of soluble epoxide hydrolase (sEH, *Ephx2* gene encoding protein) expression [6]. Given that hypermethylated and hypomethylated regions can coexist in the genome and that global DNA methylation status may not correspond to the methylation status of specific genomic regions, studies examining single-gene methylation (or histone modification) and expression may lead to a better understanding of the epigenetic effects of melatonin on programmed hypertension.

Maternal nutritional manipulations result in epigenetic regulation of specific genes [18]. Our data indicate that the potential role of melatonin in preventing programmed hypertension may be due to epigenetic regulation of genes related to RAS and nephrogenesis.

First, the RAS plays a fundamental role in the regulation of BP and kidney development. Several RAS components, including angiotensinogen (*Agt*), renin, angiotensin-converting enzyme (*Ace1*) and angiotensin II type 1 receptor (*Agtr1a*), have been reported to be epigenetically controlled via HDACs [28]. In addition, it is proposed that HDAC–RAS cross-talk contributes to ureteric bud branching during nephrogenesis [28]. Our results suggest that prenatal exposure to DEX leads to programmed expression of specific genes in the RAS via HDACs and that this can be deprogrammed by melatonin administration early in life [7]. However, the underlying epigenetic mechanisms involved in the control of RAS by melatonin require further elucidation. Second, we found that metanephroi exposed to glucose or dexamethasone exhibited low nephron numbers, which was prevented by melatonin therapy [5,7]. We also found upregulation of fibroblast growth factor 2 (*Fgf2*) and paired box gene 2 (*Pax2*) mRNA in melatonin-treated offspring [7]. Moreover, we observed that melatonin treatment up-regulated renal *Pax2* mRNA expression in the CR model. Because *Pax2* is related to congenital renal and ureteral malformations, further studies are warranted to elucidate the epigenetic effect of melatonin on *Pax2* expression and nephrogenesis.

## 4. Epigenetic Regulation of Melatonin in Normal Offspring

While melatonin therapy has a remarkably benign safety profile [14], epigenetic effects on a few key genes leading to permanent changes in nephrons (e.g., *Pax2*) and resetting of RAS may be persistent in normal adult offspring. A previous study demonstrated that maternal melatonin therapy has adverse effects on renal growth and survival in the Wistar–Kyoto rat [29]. Our recent work showed higher body weight and kidney weight in melatonin-treated offspring compared to control rats [4]. In rats, nephrogenesis occurs predominantly from late gestation to postnatal Week 1, and litters wean by postnatal Week 3. Our study was conducted using Sprague–Dawley pregnant rats, which received 0.01% melatonin in drinking water during the entire pregnancy and lactation (*i.e*., a total of six weeks), to cover the entire period of nephrogenesis [6,7]. Kidneys were subsequently collected from 1-week-, 12-week- and 16-week-old male offspring. The renal transcriptome was analyzed by whole-genome RNA next-generation sequencing (NGS).

**Figure 1:**
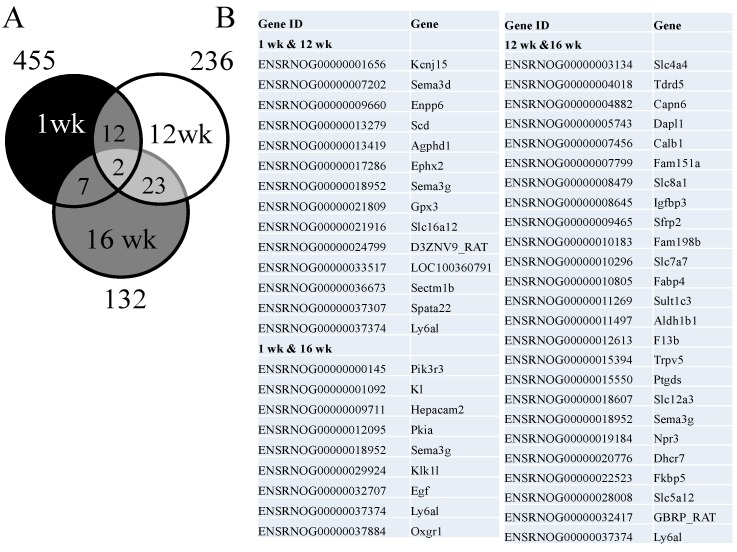
(**A**) Venn diagram depicting unique and shared (over-lapping circles) sets of differentially expressed genes (DEGs) in the kidney by maternal melatonin therapy between one week (black circle), 12 weeks (white circle) and 16 weeks of age (grey circle); (**B**) A total of 44 combined DEGs are listed.

Our first observation was that >450 genes are altered by maternal melatonin therapy in the kidney at one week of age, while these epigenetic effects become less frequent as adulthood commences. Among the differentially expressed genes (DEGs), a total of 455 genes (439 up-regulated and 16 down-regulated genes by melatonin *vs.* control at one week of age) met the selection criteria of: (1) genes that changed by reads per kilobase of transcript per million mapped reads (RPKM) >0.3; and (2) a minimum of a two-fold difference in normalized read counts between groups. Next, a total of 230 DEGs (154 up-regulated and 76 down-regulated genes) was noted in response to melatonin therapy in offspring at 12 weeks of age. In the kidney of 16-week-old offspring, there was a total of 132 DEGs (98 up-regulated and 34 down-regulated genes) between the melatonin and control groups. Genes shared by different ages are represented graphically by the Venn diagram (Figure 1). Among them, two shared genes were identified among three different developmental windows: semaphorin 3G (*Sema3g*) and lymphocyte antigen 6 complex, locus A (*Ly6al*). Interestingly, both genes are related to immune function. As shown in Table 1, we found >20 significantly related Kyoto Encyclopedia of Genes and Genomes (KEGG) pathways in the kidney of melatonin-treated offspring *vs.* control at one week of age. Similar to the changes in DEGs, the number of significant KEGG pathways decreased with age. Given the pleiotropic bioactivities of melatonin that regulate a variety of physiological functions, it is not surprising that several biological pathways are regulated by melatonin during nephrogenesis, including focal adhesion signaling, the peroxisome proliferator-activated receptors (PPAR) signaling pathway, fatty acid metabolism, the transforming growth factor (TGF)-β signaling pathway, the wingless-Int (Wnt) signaling pathway and the erythroblastic leukemia viral oncogene (*ErbB*) signaling pathway. Some pathways could be persistently regulated until adult life, such as the PPAR and *ErbB* signaling pathways.

**Table 1:** Significantly regulated Kyoto Encyclopedia of Genes and Genomes (KEGG) pathways in the kidney of maternal melatonin-treated offspring *vs.* control at different ages.

Term		Count		%		*p*-Value		Benjamini	
1 week old									
Tryptophan metabolism		9		2.1		1.2 × 10^−5^		1.6 × 10^−3^	
Pathways in cancer		18		4.1		3.5 × 10^−3^		2.1 × 10^−1^	
Focal adhesion		13		3.0		4.7 × 10^−3^		1.9 × 10^−1^	
Small cell lung cancer		8		1.8		5.7 × 10^−3^		1.7 × 10^−1^	
Vascular smooth muscle contraction		9		2.1		9.1 × 10^−3^		2.2 × 10^−1^	
PPAR signaling pathway		7		1.6		1.0 × 10^−2^		2.0 × 10^−1^	
Adherens junction		7		1.6		1.2 × 10^−2^		2.1 × 10^−1^	
Colorectal cancer		7		1.6		1.9 × 10^−2^		2.7 × 10^−1^	
Fatty acid metabolism		5		1.1		2.3 × 10^−2^		2.9 × 10^−1^	
TGF-β signaling pathway		7		1.6		2.4 × 10^−2^		2.8 × 10^−1^	
Limonene and pinene degradation		3		0.7		3.2 × 10^−2^		3.3 × 10^−1^	
Pancreatic cancer		6		1.4		3.4 × 10^−2^		3.2 × 10^−1^	
Wnt signaling pathway		9		2.1		3.5 × 10^−2^		3.1 × 10^−1^	
Endocytosis		11		2.5		3.7 × 10^−2^		3.0 × 10^−1^	
Chronic myeloid leukemia		6		1.4		4.6 × 10^−2^		3.4 × 10^−1^	
Axon guidance		8		1.8		4.8 × 10^−2^		3.4 × 10^−1^	
ECM-receptor interaction		6		1.4		6.0 × 10^−2^		3.8 × 10^−1^	
Apoptosis		6		1.4		7.1 × 10^−2^		4.2 × 10^−1^	
Lysine degradation		4		0.9		9.1 × 10^−2^		4.9 × 10^−1^	
Melanogenesis		6		1.4		9.2 × 10^−2^		4.8 × 10^−1^	
Adipocytokine signaling pathway		5		1.1		9.7 × 10^−2^		4.8 × 10^−1^	
Renal cell carcinoma		5		1.1		1.1 × 10^−1^		4.9 × 10^−1^	
Glycerolipid metabolism		4		0.9		1.1 × 10^−1^		4.8 × 10^−1^	
Ubiquitin-mediated proteolysis		7		1.6		1.1 × 10^−1^		4.8 × 10^−1^	
Biosynthesis of unsaturated fatty acids		3		0.7		1.3 × 10^−1^		5.2 × 10^−1^	
Heparan sulfate biosynthesis		3		0.7		1.5 × 10^−1^		5.6 × 10^−1^	
*ErbB* signaling pathway		5		1.1		1.8 × 10^−1^		6.3 × 10^−1^	
12 weeks old									
Complement and coagulation cascades		7		3.2		3.4 × 10^−4^		3.3 × 10^−2^	
Arachidonic acid metabolism		6		2.8		2.5 × 10^−3^		1.1 × 10^−1^	
Butanoate metabolism		4		1.8		9.8 × 10^−3^		2.8 × 10^−1^	
Systemic lupus erythematosus		5		2.3		3.3 × 10^−2^		5.7 × 10^−1^	
Nitrogen metabolism		3		1.4		3.8 × 10^−2^		5.4 × 10^−1^	
PPAR signaling pathway		4		1.8		7.2 × 10^−2^		7.0 × 10^−1^	
Chemokine signaling pathway		6		2.8		8.2 × 10^−2^		7.0 × 10^−1^	
Synthesis and degradation of ketone bodies		2		0.9		1.2 × 10^−1^		7.8 × 10^−1^	
Glutathione metabolism		3		1.4		1.5 × 10^−1^		8.2 × 10^−1^	
Natural killer cell-mediated cytotoxicity		4		1.8		1.5 × 10^−1^		8.1 × 10^−1^	
Circadian rhythm		2		0.9		1.6 × 10^−1^		8.0 × 10^−1^	
16 weeks old									
Hypertrophic cardiomyopathy (HCM)		3		2.4		1.0 × 10^−1^		1.0 × 10^0^	
*ErbB* signaling pathway		3		2.4		1.0 × 10^−1^		9.9 × 10^−1^	
Calcium signaling pathway		4		3.1		1.1 × 10^−1^		9.8 × 10^−1^	
Dilated cardiomyopathy		3		2.4		1.1 × 10^−1^		9.4 × 10^−1^	

Another observation is that several identified DEGs, including epoxide hydrolase 2 (*Ephx2*), natriuretic peptide receptor C (*Npr3*), kallikrein 1-like peptidase (*Klk1l*) and prostaglandin D2 synthase (*Ptgds*), are related to the regulation of BP. The endothelium maintains the balance between vasodilators and vasoconstrictors to control BP. The endothelium controls vascular tone using several factors causing hyperpolarization of the smooth muscle cells, namely endothelium-derived hyperpolarizing factors (EDHF). Interestingly, most identified DEGs related to BP control belong to EDHFs. In addition to NO, our previous study suggests that other EDHFs, namely arachidonic acid metabolites, might play a role in programmed hypertension [6]. Given that normal gestation and pregnancy require a finely-tuned vasodilator/vasoconstrictor balance, the question of whether maternal melatonin therapy may permanently alter the balance of EDHFs leading to deficient adaptations and fetal programming in later life awaits further evaluation.

We next used our NGS dataset to investigate five groups of epigenetic regulators, encoding DNMTs, HDACs, histone methyl- and acetyl-transferase, bromodomain-containing proteins recognizing acetylated lysine residues in histone tails and chromodomain-containing proteins recognizing methylated histones (present in the RNA-induced silencing complex) [30]. As shown in Table 2, melatonin up-regulates several epigenetic regulator genes during nephrogenesis, including DNA methyltransferase 3A (*Dnmt3a*), histone deacetylase 4 (*Hdac4*), histone deacetylase 7 (*Hdac7-rat*), histone deacetylase 1-like (*Hdac1l*), chromodomain helicase DNA binding protein 1 (*Chd1*), *Chd2*, *Chd3*, bromodomain and PHD finger containing 3 (*Brpf3*), tyrosine-protein kinase BAZ1B (*Baz1b*) and bromodomain and WD repeat domain containing 2 (*Wdr11*). However, at 16 weeks of age, melatonin only downregulates *Dnmt3b* and up-regulates DNA (cytosine-5)-methyl-transferase 3-like (*Dnmt3l*) and *Hdac4*.

**Table 2:** Changes of epigenetic regulator genes in the kidney of melatonin-treated offspring *vs.* control at one week and 16 weeks of age.

			1 Week			16 Weeks		
Gene ID	Gene Symbol	Description	Control	Melatonin	Fold Changes	Control	Melatonin	Fold Changes
*ENSRNOG00000039859*	*DNMT1_RAT*	*DNA methyltransferase 1*	2.051	3.705	1.81	1.277	1.615	1.27
*ENSRNOG00000026132*	*Trdmt1*	*DNA methyltransferase 2*	2.027	2.970	1.46	2.316	4.001	1.73
*ENSRNOG00000026649*	*Dnmt3a*	*DNA methyltransferase 3A*	2.140	4.859	**2.27**	1.372	1.424	1.04
*ENSRNOG00000010625*	*Dnmt3b*	*DNA methyltransferase 3B*	2.082	1.546	0.74	0.346	0.106	**0.31**
*ENSRNOG00000001212*	*Dnmt3l*	*DNA (cytosine-5)-methyl-transferase 3-like*	0.349	0.130	**0.37**	0.127	0.425	**3.35**
*ENSRNOG00000009568*	*Hdac1*	*histone deacetylase 1*	41.010	43.771	1.07	31.233	27.163	0.87
*ENSRNOG00000000604*	*Hdac2*	*histone deacetylase 2*	61.325	46.276	0.75	31.843	31.081	0.98
*ENSRNOG00000019618*	*HDAC3_RAT*	*histone deacetylase 3*	22.270	20.865	0.94	13.929	12.779	0.92
*ENSRNOG00000020372*	*Hdac4*	*histone deacetylase 4*	0.241	0.794	**3.30**	0.390	0.858	**2.20**
*ENSRNOG00000020905*	*Hdac5*	*histone deacetylase 5*	18.740	14.185	0.76	7.330	6.855	0.94
*ENSRNOG00000006791*	*Hdac6*	*histone deacetylase 6*	13.085	16.147	1.23	31.671	15.609	0.49
*ENSRNOG00000008308*	*HDAC7_RAT*	*histone deacetylase 7*	2.607	5.539	**2.12**	0.754	1.039	1.38
*ENSRNOG00000003122*	*Hdac8*	*histone deacetylase 8*	3.894	4.662	1.20	5.390	4.028	0.75
*ENSRNOG00000004158*	*Hdac9*	*histone deacetylase 9*	0.063	0.098	1.55	0.148	ND	ND
*ENSRNOG00000031915*	*Hdac10*	*histone deacetylase 10*	1.619	0.999	0.62	1.085	1.011	0.93
*ENSRNOG00000006824*	*Hdac11*	*histone deacetylase 11*	6.602	9.214	1.40	10.496	9.769	0.93
*ENSRNOG00000013695*	*Hdac1l*	*histone deacetylase 1-like*	0.116	0.598	**5.14**	0.221	0.284	1.29
*ENSRNOG00000014434*	*Chd1*	*chromodomain helicase DNA binding protein 1*	1.031	3.086	**2.99**	2.707	4.965	1.83
*ENSRNOG00000012716*	*Chd2*	*chromodomain helicase DNA binding protein 2*	1.072	2.489	**2.32**	4.534	6.844	1.51
*ENSRNOG00000009722*	*Chd3*	*chromodomain helicase DNA binding protein 3*	4.460	10.610	**2.38**	5.264	6.224	1.18
*ENSRNOG00000018309*	*Chd4*	*chromodomain helicase DNA binding protein 4*	11.267	17.581	1.56	13.285	14.230	1.07
*ENSRNOG00000011268*	*Chd5*	*chromodomain helicase DNA binding protein 5*	0.018	ND	ND	0.060	ND	ND
*ENSRNOG00000025011*	*Chd8*	*ATP-dependent helicase CHD8*	3.620	3.711	1.03	2.860	2.958	1.03
*ENSRNOG00000004538*	*Brd1*	*bromodomain containing 1*	16.720	13.148	0.79	12.115	13.531	1.12
*ENSRNOG00000000461*	*Brd2*	*bromodomain-containing 2*	18.545	27.024	1.46	26.034	27.635	1.06
*ENSRNOG00000006770*	*Brd4*	*bromodomain containing 4*	5.530	5.022	0.91	5.116	7.069	1.38
*ENSRNOG00000014419*	*Brd7*	*bromodomain containing 7*	28.057	24.411	0.87	17.878	16.157	0.90
*ENSRNOG00000020340*	*Brd8*	*bromodomain containing 8*	7.651	8.557	1.12	7.289	7.010	0.96
*ENSRNOG00000015676*	*Brd9*	*bromodomain containing 9*	11.196	10.334	0.92	7.379	8.071	1.09
*ENSRNOG00000028641*	*Brpf3*	*bromodomain and PHD finger containing 3*	1.375	3.167	**2.30**	1.585	1.127	0.71
*ENSRNOG00000001453*	*Baz1b*	*tyrosine-protein kinase BAZ1B*	2.262	5.310	**2.35**	5.829	7.782	1.34
*ENSRNOG00000025148*	*Baz2b*	*bromodomain adjacent to zinc finger domain protein 2B*	1.323	2.093	1.58	2.281	2.865	1.26
*ENSRNOG00000002073*	*Brdt*	*bromodomain, testis-specific*	0.162	0.316	1.95	0.245	0.415	1.69
*ENSRNOG00000001632*	*Brwd1*	*Bromodomain and WD repeat domain containing 1*	1.323	1.865	1.41	2.330	4.132	1.77
*ENSRNOG00000020430*	*Wdr11*	*bromodomain and WD repeat domain containing 2*	2.032	5.116	**2.52**	6.643	6.917	1.04
*ENSRNOG00000002291*	*Brwd3*	*bromodomain and WD repeat domain containing 3*	0.559	0.770	1.38	1.010	1.594	1.58
*ENSRNOG00000028816*	*Baz2a*	*bromodomain adjacent to zinc finger domain, 2A*	1.161	2.261	1.95	2.722	2.556	0.94
*ENSRNOG00000019585*	*Myst1*	*histone acetyltransferase KAT*	7.576	7.963	1.05	10.915	7.914	0.73
*ENSRNOG00000022664*	*Myst2*	*Kat7*	6.592	7.865	1.19	8.121	6.860	0.84
*ENSRNOG00000025174*	*Myst3*	*histone acetyltransferase KAT6A*	3.329	4.329	1.30	3.916	5.116	1.31
*ENSRNOG00000007242*	*Ehmt1*	*H3 lysine-9 specific 5*	6.873	6.275	0.91	2.692	2.816	1.05
*ENSRNOG00000030630*	*Ehmt2*	*H3 lysine-9 specific 3*	14.084	15.443	1.10	9.394	8.454	0.90
*ENSRNOG00000001524*	*Hat1*	*histone acetyltransferase 1*	20.997	32.990	1.57	28.149	32.895	1.17

Quantification for gene expression was calculated as reads per kilobase of exon per million mapped reads (RPKM). Genes that changed by RPKM >0.3- and ≥2-fold differences between melatonin-treated offspring *vs*. control are indicated in bold type. ND = not detectable.

It is noteworthy that six weeks of maternal melatonin therapy is likely to upregulate, but not down-regulate, genes in the offspring kidney. In agreement with previous studies [4,13], our findings suggest melatonin may serve as an inducer of gene expression in the developing kidney. Our data also indicate that epigenetic changes associated with programming by early melatonin therapy may disappear during the course of development and that differential patterns of epigenetic regulation may occur during different developmental windows.

## 5. Are We Ready to Apply Melatonin in Clinical Practice to Prevent Programmed Hypertension?

So far, there are some major blocks to the clinical application of melatonin to prevent programmed hypertension. First, identification of patients at risk of developing programmed hypertension remains impracticable. Patients who are at risk should be closely followed throughout life. Preterm birth and low birth weight (LBW) are risk factors for the development of programmed hypertension in later life [31]. Despite recent advances in the elucidation of the underlying mechanisms linking programming processes, our understanding of clinical surrogate markers to identify patients at risk is still too limited. Currently, the available surrogate markers for low nephron number include LBW, intrauterine growth retardation (IUGR), short stature and reduced kidney volume on ultrasound imaging [32]. However, most markers are not specific, and programmed hypertension could be dissociated from a low nephron number; Second, patients with prehypertension or at risk for other BP abnormalities should be assessed by 24-h ambulatory blood pressure monitoring (ABPM) instead of office BP. Nevertheless, measurements of melatonin level and 24-hour ABPM in patients are not yet performed on a routine basis; Third, the long-term effects of melatonin on neonates remain unknown. While melatonin has been shown to reduce oxidative stress in neonates with sepsis, asphyxia, respiratory distress and surgical stress in some small-scale trials [33], further, large, multicenter collaborations are required to conduct meaningful clinical research studies to explore the safety and efficacy of melatonin in clinical practice.

## 6. Conclusions

In conclusion, this review provides an overview of experimental approaches investigating the epigenetic regulation of melatonin in programmed hypertension, with special emphasis on the kidney: (1) it discusses the role of melatonin in epigenetic regulation, such as DNMT and HDAC; (2) it presents a series of studies that demonstrate the beneficial effects of melatonin on programmed hypertension; (3) it demonstrates the long-term epigenetic effects of maternal melatonin therapy in the normal offspring kidney by NGS analysis; and (4) it indicates problems that must be addressed before melatonin use can be translated into clinical practice to prevent programmed hypertension.

Whereas there has been extensive study of aspects of melatonin in treating established hypertension [34,35,36], there has been little recent investigation into epigenetic regulation of melatonin on programmed hypertension, which is surprising, since early intervention can have a profound effect in reducing the future burden of hypertension. Recent experimental evidence has shown that melatonin is able to epigenetically regulate specific genes and pathways in the kidney to prevent programmed hypertension. Whether early melatonin therapy might cause long-term epigenetic changes leading to adverse effects in adulthood, however, remains to be elucidated.
